# Sociodemographic Correlates of Tobacco Consumption in Rural Gujarat, India

**DOI:** 10.1155/2016/5856740

**Published:** 2016-03-30

**Authors:** Payal Kahar, Ranjita Misra, Thakor G. Patel

**Affiliations:** Department of Social & Behavioral Sciences, Robert C. Byrd Health Sciences Center, School of Public Health, West Virginia University, 3313A, Morgantown, WV 26506-9190, USA

## Abstract

*Background.* The purpose of this study was to examine occupation-, education-, and gender-specific patterns of tobacco use and knowledge of its health effects among 23,953 rural Asian Indians ≥18 years in Gujarat.* Methodology.* A statewide, community-based, cross-sectional survey was conducted in 26 districts of Gujarat (December 2010–May 2015), using face-to-face interviews by trained community health workers called SEVAKS.* Results.* Mean age was 39.8 ± 15.2 years. Eighteen percent of respondents used tobacco in various forms. Tobacco consumption was significantly higher among males (32%), 18–34 years' age group (35%), those who were self-employed (72%), and those with elementary education (40%). The prevalence was 11 times higher among males than females (95% CI = 9.78, 13.13). Adjusted ORs for tobacco use showed strong gradient by age and educational level; consumption was lower among the illiterates and higher for older participants (≥55 years). Tobacco consumption also varied by occupation; that is, those who were self-employed and employed for wages were more likely to use tobacco than those who were unemployed. Knowledge of health effects of tobacco lowered the odds of consumption by 30–40%.* Conclusions.* Effective educational programs should be tailored by gender, to improve knowledge of health risks and dispel myths on perceived benefits of tobacco.

## 1. Introduction

It has been long documented that tobacco use contributes to high rates of morbidity and mortality across the world [1]. Not only is it a major risk factor for various types of chronic diseases such as cancers, chronic obstructive pulmonary diseases, cardiovascular diseases, and poor reproductive outcomes, but also it is associated with high rates of dental diseases such as dental caries, periodontal diseases, and tooth loss [2]. In spite of great achievements in public health globally, problems with tobacco consumption exist in both developing and developed nations. In South Asian countries such as India, the burden of oropharyngeal cancers and dental problems (e.g., teeth attrition, periodontitis, and premature tooth loss) is high [1, 3]. Tobacco consumption is responsible for 50% of all the cancers in men and 25% in women [4].

In India, tobacco is consumed in several forms, which include smoking as well as smokeless tobacco. While “bidis,” which are small, thin hand-rolled cigarettes comprising of tobacco wrapped in tendu leaves, are predominantly smoked in rural India, other available varieties are hookah, chuttas, cigarettes, and cigars [5]. Tobacco is also chewed extensively in India; use of chewable tobacco is in the form of paan masala, gutka, and other locally prepared mixtures of tobacco, areca nut, and additives. Paan is made from piper betel leaf filled with sliced areca nut, lime, catechu, and other spices chewed with or without tobacco [6]. Chewing of paan with tobacco is a popular habit that has been integrated into customs and traditions in rural India [7].

Habitual chewing of betel squid or use of tobacco in smoking or smokeless forms by men and women in India is due to less awareness of its health hazards or because of prevalent sociocultural perceptions of its beneficial effects [7]. Some of the existing myths, especially among rural Indians, about the benefits of tobacco use include the following: it relieves anxiety and tension, induces feeling of pleasure, and decreases orodental pain and swelling [7]. In addition, social norms, availability, acceptability, and advertising campaigns also influence tobacco use, particularly among males [8]. Hence, gender disparities in tobacco use are noted with a significantly higher proportion of males and low-income people consuming tobacco in one or several forms [9]. Previous studies show that tobacco consumption is higher among the less educated, older age groups (especially middle-aged males), and agricultural and labor workers [6, 9].

According to the recent Global Adult Tobacco Survey (GATS, 2009-2010), one-third of Indian population ≥15 years of age use tobacco in some form [6]. Indian males over 15 years of age are twice more likely to use tobacco (47.9%) as compared to their female peers (20.3%). While statewide, population-based research on the role of poor health, rurality, and socioeconomic factors in disparities of tobacco consumption is limited, the GATS survey showed that in the state of Gujarat 20.1–30% of population use tobacco [6]. Gujarat is one of the most progressive states located in western India and has a population of 60.3 million (5% of the Indian population). Over half of the population in Gujarat or 57.4% live in the rural areas [10]. Despite a surge of epidemiological studies assessing the prevalence and determinants of tobacco use in the state of Gujarat, most have focused on urban populations or localized to one or more districts within the state [11, 12]. There is a dearth of studies that have examined the prevalence and sociodemographic predictors of tobacco use in rural regions of Gujarat.

This study uses data from the SEVAK project (Sanitation and Health Education in Village Communities through Improved Awareness and Knowledge of Prevention/Management of Diseases and Health Promotion), a statewide chronic disease prevention and management intervention that was launched in 2010. The SEVAK project provided a representative sample of rural Gujaratis in remote regions in all the 26 districts of the state. The overall goal of the project was to create standardized delivery of diabetes, obesity, and hypertension screening and access to care of rural Indians by trained community health educators called SEVAKS. SEVAKS were trained in culturally and linguistically appropriate data collection using face-to-face interviews on behavioral risk factors, environmental factors, and chronic diseases including tobacco consumption in various forms of participants. Hence, the purpose of this study was to (1) determine the prevalence of tobacco use by gender; (2) assess their knowledge of harmful health effects; and (3) examine sociodemographic predictors of tobacco use (overall, smoking, and chewing/smokeless form).

## 2. Methods

### 2.1. Research Design and Procedure

This was a statewide cross-sectional study conducted in all the 26 districts of the state. Twenty-six trained SEVAKS collected data via face-to-face interviews using standardized survey questionnaire in local language that was developed for the project. The tobacco use question came from Behavioral Risk Factor Surveillance System (Centers for Disease Control and Prevention, United States) [13], and the questions on various forms were added as a checklist with frequency of use and types.

Items on the type and the frequency of tobacco use are provided in Tobacco Use and Knowledge.

### 2.2. Participants

A total of 23,953 participants completed the survey questionnaire (response rate ~94%). The sample was large enough to provide reliable estimates of prevalence of tobacco consumption by sex and socioeconomic population groups. Participants are comprised of adults over 18 years of age and residing in rural regions of the state. Individuals younger than 18 years and adults whose medical conditions did not allow accurate assessments were excluded from the study. Data collection occurred between December 2010 and May 2015.

### 2.3. Measures

#### 2.3.1. Demographics

Participants' demographic information included age, gender, educational status, and income level. Age, collected in years, was treated as a categorical variable and subdivided into four groups: 18–34 years, 35–44 years, 45–54 years, and 55 years and above.

#### 2.3.2. Tobacco Use and Knowledge

The survey had several questions on tobacco use: participants were asked if they currently used tobacco in any form and those who responded “yes” were asked probing question on the forms of use. For those who used cigarettes and bidis, probes included use “every day,” “some days,” or “not at all.” For all other forms such as hookah, gutka, paan, and chewing tobacco and tobacco paste, participants could check “yes” or “no” and reported the frequency of use and consumption of the various tobacco products. Participants had the option to choose multiple forms of tobacco usage. Answers were collapsed to report on the various forms (Table 1).

Two questions elicited information on participants' knowledge regarding the health hazards of tobacco use. (1) Does smoking cause lung and other cancers? (2) Do chewing tobacco and/or smoking cause mouth cancer? Each correct response to these questions was given a value of “1” and incorrect response a value of “0.” The knowledge score was calculated by summing the correct responses to the two cancer awareness questions (range 0–2). Cronbach's alpha for the knowledge score was 0.75, indicating good reliability.

### 2.4. Data Analysis

Completed data collection forms were deidentified and data were coded and entered into an SPSS database (SPSS Inc., Chicago, IL). Descriptive statistics included gender, age, income, educational level, and tobacco use. Point estimates of prevalence of tobacco consumption were calculated. Analysis was also conducted to detect the differences in health hazard of tobacco consumption knowledge by age, occupation, and educational level; distribution of tobacco consumption by demographic characteristics was analyzed using Chi-square. Finally, logistic regression was used to estimate the influence of sociodemographic factors and knowledge level on tobacco consumption (overall, smoking, and smokeless/chewing tobacco) by users and nonusers.

## 3. Results

The total number of participants who had completed the surveys between December 2010 and May 2015 was 23,953. The mean age of the sample was 39.78 ± 15.23 years (range 18–88 years). The majority or approximately 40% of the participants were between 18 and 34 years of age followed by 35–44 years' (21.7%) age group and 36.2% who were 45 years old and older (Table 1). The sample was almost evenly distributed by gender, that is, males (51.7%) and females (48.3%). Information on the occupational status of participants showed that 43% were self-employed (as farmers or small business owners) and approximately half reported that they were unemployed. In addition, the majority of females were homemakers and only a few (0.6%) were employed for wages. Rural Indians tend to have higher levels of illiteracy and our study reflected that approximately one-third had no formal education, 39% reported elementary level education, 22% had some high school education or were high school graduates, and only 3% had a college degree.

### 3.1. Overall Prevalence of Tobacco Consumption

The overall prevalence of tobacco use (in all forms) was 18.2%; among all participants, 14.2% smoked while 9.4% chewed tobacco in several forms. Among tobacco users, 77.8% smoked bidis and cigarettes while 51.7% chewed or used smokeless forms of tobacco such as paan masala, paan, gutka, toothpaste, and other commercially available tobacco products. A higher frequency of tobacco consumption, that is, between 8 and 10 times a day, and in various forms, was reported by several participants in our study. Approximately 27% of male and female respondents in rural Gujarat used tobacco in both forms of smoking and chewing/smokeless tobacco.

### 3.2. Knowledge of Health Hazards of Tobacco Consumption

The knowledge of health hazards associated with tobacco consumption was compared for males and females across age, occupation, educational level, and type of tobacco use (Table 2). Overall, rural males had significantly higher knowledge of the health consequences associated with tobacco use than rural females (*p* < .001). In addition, younger individuals between the ages of 18–34 years had significantly higher knowledge (1.74 ± 0.59) than individuals over 35 years of age (*F* statistic = 47.8; *p* < .001). Comparison of tobacco knowledge by occupational status showed that self-employed individuals had significantly higher knowledge (1.76 ± 0.58) than those who reported that they were unemployed or employed for wages (*F* statistic = 49.9; *p* < .001). Participants with elementary and high school education had higher knowledge (1.75 ± 0.58; 1.76 ± 0.55, resp.) than those with no formal education. Interestingly, they also had higher knowledge of the health hazards of tobacco use than those who reported that they had college education (*F* statistic = 49.9; *p* < .001). Overall, nonusers had higher awareness of the health hazards of tobacco use than tobacco users (1.75 ± 0.59; *F* statistic = 49.9; *p* < .001).

### 3.3. Tobacco Consumption by Socioeconomic Variables

Table 3 shows tobacco consumption by gender, age, occupation, and educational levels. Significantly more males (32.2%) than females (3.3%) consumed tobacco in various forms. Self-reported use of tobacco was the highest among young adults, 15–34 years of age (35%), and this differential pattern was statistically significant across age categories (*χ* ^2^ = 117.25; *p* < .001). Individuals who were self-employed (76.7%) were more likely to use tobacco than individuals who were unemployed (20.6%) or employed for wages (7.1%); the association between tobacco consumption and occupation was statistically significant (*χ* ^2^ = 2583.4; *p* < .001). Likewise, participants with elementary and high school education used tobacco (39.5% and 33%, resp.) more than individuals with no formal education (23.2%) and with a college degree (4.3%). The association between tobacco use and educational categories was found to be statistically significant (*χ* ^2^ = 562.02; *p* < .001).

### 3.4. Socioeconomic Predictors of Tobacco Consumption

Binary logistic regression was performed with tobacco use (overall, smoking, and smokeless tobacco) as the dependent variable and demographic variables such as gender, age, occupation, educational level, and knowledge score as the predictor variables. Bivariate unadjusted and multivariate adjusted odds ratios (ORs) are shown in Table 4. The results between the unadjusted and adjusted ORs were consistent for all variables and were significant. As shown in the adjusted model, overall, rural males were 11 times more likely to consume tobacco than females (OR = 11.33; 95% CI = 9.78, 13.13). This increased for smoking cigarettes and bidis (OR = 24.32; 95% CI = 19.91, 29.70) but decreased slightly for smokeless and chewing tobacco (OR = 9.67; 95% CI 7.87, 11.89). Similarly, older individuals had twice the odds of using tobacco compared to individuals aged 18–34 years, showing higher proportion of older people consumed tobacco in one or more forms, and tobacco consumption increased with age. Participants who were employed for wages also had a higher likelihood of consuming tobacco in various forms (OR = 2.16 for overall use and 1.12 and 1.82 for smoking and chewing/smokeless tobacco, resp.) than those who were self-employed while unemployed individuals had 17 to 30% lower odds of consuming tobacco in various forms. Participants who were considered educated in rural areas, that is, with a high school and/or a college degree, had 2.4 times higher odds of consuming tobacco but were 56% less likely to smoke and preferred the use of chewing and smokeless tobacco (OR = 3.70; 95% CI = 2.91, 4.70) as compared to participants with no formal education. When comparing those with no formal education, participants with elementary education had 1.7 times higher odds of consuming tobacco (overall and chewing/smokeless tobacco) but not for the use of cigarettes and bidis (Table 4). Finally, individuals with higher knowledge of health consequences of tobacco use were less likely to use it; a unit increase in knowledge resulted in lowering the odds of tobacco consumption in all forms by 40% and chewing/smokeless tobacco use by 30%. However, individuals who smoked had higher knowledge of the harmful effects of tobacco use (OR = 1.38; 95% CI = 1.29, 1.47).

## 4. Discussion

To the best of our knowledge, this is the first study that examined tobacco knowledge and sociodemographic predictors of tobacco use in the rural regions of Gujarat, India. Our findings confirm overall prevalence of 18.2%, but the prevalence rate was lower than the state average of 29.4% among Gujaratis aged ≥15 years as reported by the GATS 2009-2010 India survey [6]. Furthermore, the smokeless tobacco consumption (9.4%) was below the state prevalence of 20.1–30%, while smoking (14.2%) paralleled the reported state range of 10.1–20% [6]. It can be definitely speculated that the lower prevalence rates in our study may be due to underreporting due to social desirability as the community health workers live in their communities. Furthermore, assessments of their tobacco use were part of an overall survey questionnaire that asked about behavioral and environmental risk factors for chronic conditions. Hence, there could be additional subjective bias towards more social desirable answers. Other factors that could be accounted for a lower prevalence rate are as follows: participants may consider themselves to be light users, trying to quit, and lie about their use due to fear of repercussions [14]. Additionally, the variation in our results to reported prevalence may be due to the differences in participants' age; that is, individuals, 15 years old and older, were included in GATS survey while participants were adults, 18 years of age and older, in our study. Hence, this may have contributed to our lower prevalence of chewing tobacco since prevalence of chewing tobacco was found to be 10% for 13–17-year-olds and 51.3% among 17–19-year-olds in urban Gujarat [11]. While differential patterns in tobacco use may exist by urban and rural areas, a large proportion of the adolescents below the age of 18 years use tobacco that was not captured by our study. Other probable factors for lower prevalence rates in this study might be due to our use of raw and observational data as compared to weighted sampling and multistage clustered design in larger studies such as GATS survey [11]. In addition, the GATS survey also excluded the institutionalized population and extreme remote areas from the target population. Since this study collected data from remote and rural communities in Gujarat, our study population may have been different from other reported studies and could explain the lower overall prevalence rate among women. While our prevalence rates were lower than the statewide rate as reported in the GATS Gujarat survey and others, significant discrepancy in smoking prevalence has also been reported in various statewide tobacco surveillance studies [6, 12]. For example, in the United States, a 10% lower prevalence rate of tobacco use was noted in the Adult Tobacco Survey as compared to the Behavioral Risk Factor Surveillance System in West Virginia [15].

Tobacco is used for several recreational and therapeutic reasons [16–18] and tobacco-based dentifrices are believed to be germicidal aiding in effective teeth cleaning while reducing pain due to dental problems [19, 20]. While stressors or peer pressure is noted for tobacco initiation, cultural practices that promote tobacco behavior include use for social interaction (a sense of camaraderie) and symbolic functions (so as to celebrate wedding rituals and religious ceremonies) and extending hospitality to family and friends [21, 22]. Hence, educational programs should be tailored to change the deep-rooted cultural beliefs and myths that tobacco aids in digestion (paan), acts as mouth freshener (gutka, paan masala), and relieves anxiety, stress, and orodental pain. Prevention efforts should include harmonizing tobacco tax and prices, empowering decision-makers and policymakers, and community opinion/local leaders for primary prevention and reduce tobacco-related death and disability.

Tobacco products such as bidis, gutka, and paan masala are locally manufactured, inexpensive, and easily available in rural India. Crude forms of tobacco are easily available and accessible, are relatively cheaper, and are used by socioeconomically disadvantaged people. However, they have more harmful health effects [9, 23]. For example, bidis have higher concentrations of tar and carbon monoxide [24] and crude smokeless forms are highly carcinogenic contributing to increasing rates of oral cancers in India [25]. The socioeconomic burden of tobacco and related diseases is significantly higher than the contribution by the tobacco industry to government revenues [19]. Yet, aggressive marketing by transnational tobacco companies has expanded their markets and increased rates of consumption [26]. Furthermore, cultural acceptability and perceptions of safe and beneficial effects have resulted in greater use of smokeless and chewing tobacco, especially among women. Rural females in Gujarat tend to be light users and use tobacco in the forms of snuff, gutka, and tobacco toothpaste [12, 21]. However, a very few female participants in this study used tobacco.

Significantly lower consumption by females emphasizes the gender disparity in tobacco use in rural areas [27–30]. For example, Bala et al. assessed tobacco prevalence in four districts of Gujarat and reported prevalence of 26.5% among females [12]. Likewise, the GATS survey for the state of Gujarat, 2009-2010, reported that 11.4% of Gujarati females were tobacco users (0.3% smoked, 9.9% used smokeless tobacco, and 1.2% used both forms) [11]. A study in Anand District of Gujarat reported prevalence rates of 15.2% among rural females aged 20–69 years. The study also found higher prevalence of smokeless tobacco (14.8%) as compared to smoking tobacco (0.5%) [30]. Lower self-reported use of tobacco, especially smokeless form among females in our study, raises concerns about a social desirable bias as mentioned earlier. The social stigma associated with tobacco use among females in remote and rural areas may also have influenced their response [9].

Tobacco consumption was generally higher among educated and employed individuals. A differential pattern in usage, noted between the bivariate and multivariate analysis, may be due to educated and agricultural workers, who were predominantly males and had higher rates of consumption than females. Smokeless tobacco was preferred among older men and bidis and cigarettes were preferred among younger men. Our results are also comparable to the findings of previous local and national studies that show that males and agricultural/labor class workers used tobacco more frequently in India [6, 9, 31].

A higher use of tobacco among older adults may also predispose them to chronic diseases, as it has been shown that tobacco is a modifiable risk for noncommunicable diseases for heart disease and type 2 diabetes and its complications [32]; these chronic conditions are high among Indians [32, 33], which is the leading cause of death and disability [24] and direct and indirect health care costs [24]. The higher rates of tobacco use among the older age groups may be due to its addictive nature after initiation and cultural acceptance of its use over time. Hence, efforts to educate and reduce tobacco consumption among this group should be a public health priority.

Higher levels of knowledge linking use of tobacco with cancer concur with a study in Kerala, also a progressive state like Gujarat [8]. Other studies have shown that people in rural areas have limited knowledge about the health hazards, especially for smokeless tobacco [8, 19, 34]. Results from the Tobacco Control Policy study in rural areas of Maharashtra and Bihar showed that overall knowledge was the lowest regarding stroke and CHD (coronary heart disease) while awareness of stained teeth and mouth cancer was the highest among smokers [35]. Overestimation of knowledge may have been possible in this study as one of the two questions asked respondents whether smoking causes lung and other cancers or not. Association of smoking to lung and mouth cancers is well known as compared to its linkages with other cancers [36, 37]. Knowledge was higher among males, nonusers, and younger and self-employed individuals. Interestingly, smokers were knowledgeable about the dangers of tobacco. Overall, nonusers had higher knowledge that highlights the success of educational campaigns. Knowledge reduced the likelihood of tobacco use by 30 to 40% and concurs with prior studies showing a reduction in tobacco initiation and behavior [6, 9]. Hence, effective educational messages and programs need to be tailored for rural communities and tailored for age and gender of users since forms of use vary by these groups. Educational campaigns and warning labels can improve knowledge and use of multiple methods of communications such as TV and radio programs, folk dramas, films, exhibits, and group meetings can be beneficial. Additionally, dispelling myths and making health messages personally relevant by tailoring it to address individuals' beliefs and myths can reduce prevalence and initiation; for example, tobacco cannot cure toothache, aggravate other diseases such as diabetes and heart disease, cause cancer of the food pipe (chewing tobacco), and may harm the child during pregnancy and treatment/management of diseases due to tobacco consumption may be expensive. Effective and graphic warning labels can also create awareness about their harmful effects.

Use of health education programs and campaigns in rural areas with the use of community health workers or SEVAKS can be particularly effective as it is a bottom-up educational program to improve awareness about the health hazards and reduce initiation and/or tobacco use [6, 9]. A promising approach for effective educational campaign should target adolescent and young adults, especially males, prior to their initiation, since knowledge of its harmful effects after initiation does not always promote cessation due to its addictive nature. Furthermore, educational programs should highlight the risks and complications associated with smoking as well as smokeless/chewing tobacco among young adults as well as individuals with diabetes and multimorbidity [38–40].

Tobacco consumption is a public health challenge in India. It requires a multifaceted approach and strategies for intervention, especially in rural areas where more than 50% of its people live [41]. Due to a shortage of health care providers in rural communities, use of lay health workers (such as the SEVAKs) for educational campaigns may be cost-effective in rural areas. Finally, raising tobacco taxes and enforcing laws restricting sales to younger people below 18 years may reduce usage; the public smoking ban of 2008 has not been enforced consistently [42] and stringent laws to punish violators in rural areas are yet to be seen. The pictorial health warning labels on tobacco products, the most cost-effective tool for educating smokers about health risks, were introduced in 2009. However, graphic warning is not strictly followed, particularly in locally manufactured dentifrices such as toothpaste and toothpowders. A 1992 Government of India regulation prohibits addition of tobacco in dentifrices. Yet, many manufacturers continue to add it without warning labels [7, 43]. Whereas various regulatory policies and their enforcement have been made in recent years, a stronger and comprehensive legislation to reduce tobacco-related disparities in rural India is recommended.

Some inherent limitations in the study should be noted. The cross-sectional study design did not allow assessment of tobacco consumption over time [44]. Further, participants may have answered questions in a socially desirable manner underestimating the prevalence reported in this study. Similarly, use of tobacco in various forms and their frequency may have been underestimated. Overestimation of the knowledge among participants is also possible due to combining lung and mouth cancer with other forms of cancer. While the use of SEVAKS for data collection and their credibility in communities resulted in a high level of response and completed surveys, their familiarity with participants may have contributed to the social desirability response. Notwithstanding these limitations, the present study provided statewide prevalence of tobacco consumption in remote rural areas of the state. Future research should compare rural with urban and semiurban areas and also test the impact of interventions and educational campaigns.

## 5. Conclusion

The results from this study indicate a gender disparity in tobacco prevalence with higher usage among males, especially self-employed and low educated individuals in rural Gujarat. Effective education programs that are gender specific and community based that focus on improving awareness and knowledge of associated health risks among users using a grounds-up model by utilizing community health workers or SEVAKS are suggested. Additionally, debunking myths on perceived benefits of tobacco use as well as regulatory enforcement of warning labels on all tobacco products can reduce tobacco use and its related health and economic burden.

## Figures and Tables

**Table 1:** Demographic characteristics and knowledge of health hazards of tobacco consumption.

Variable	Male *N* (%)	Female *N* (%)	Total *N* (%)
Age			
18–34 years	5078 (41.7)	4846 (42.7)	9924 (42.2)
35–44 years	2607 (21.4)	2488 (21.9)	5095 (21.7)
45–54 years	2070 (17.0)	1878 (16.5)	3948 (16.8)
Above 55 years	2412 (19.8)	2150 (18.9)	4562 (19.4)
Mean ± SD	39.90 ± 15.08 years	39.60 ± 15.31 years	39.78 ± 15.23 years
Income level			
Employed for wages	582 (4.7)	66 (0.6)	648 (2.7)
Not employed	1943 (15.7)	11074 (95.8)	13017 (54.3)
Self-employed	9860 (79.6)	420 (3.6)	10280 (42.9)
Educational level			
No formal education	3551 (28.7)	5047 (43.7)	8598 (35.9)
Elementary education	4939 (39.9)	4397 (38.0)	9336 (39.0)
High school education	3396 (27.4)	1907 (16.5)	5303 (22.1)
Some college and college graduate	496 (4.0)	208 (1.8)	704 (2.9)
Use of tobacco in any form			
Yes	3988 (32.2)	377 (3.3)	4365 (18.2)
No	8403 (67.8)	11185 (96.7)	19588 (81.8)
Smoking (bidis/cigarettes)			
Every day	2452 (19.8)	108 (0.9)	2560 (10.7)
Some days	783 (6.3)	55 (0.5)	838 (3.5)
Not at all	9156 (73.9)	11399 (98.6)	20555 (85.8)
Chewing tobacco/use of smokeless tobacco			
Yes	2103 (17.0)	154 (1.3)	2257 (9.4)
No	10288 (83.0)	11408 (98.7)	21696 (90.6)
Knowledge regarding health risks of tobacco use			
Does smoking cause lung and other cancers?			
Yes	10329 (84.6)	9302 (81.8)	19631 (83.3)
No	1874 (15.4)	2066 (18.2)	3940 (16.7)
Does chewing tobacco/smoking cause mouth cancer?			
Yes	11111 (91.1)	10032 (88.3)	21143 (89.7)
No	1089 (8.9)	1332 (11.7)	2421 (10.3)

**Table 2:** Knowledge of tobacco hazards by gender, age, occupation, educational levels, and tobacco users versus nonusers.

Variable	Male mean (SD)	Female mean (SD)	*F* value (*p* value)
Age			47.8 (<.001)
18–34 years	1.77 (.55)	1.72 (.62)	
35–44 years	1.73 (.60)	1.68 (.66)	
45–54 years	1.75 (.58)	1.69 (.66)	
Above 55 years	1.76 (.58)	1.69 (.66)	
Occupation			49.9 (<.001)
Not employed	1.73 (.60)	1.71 (.64)	
Self-employed	1.76 (.57)	1.57 (.76)	
Employed for wages	1.70 (.57)	1.23 (.85)	
Educational levels			49.9 (<.001)
No formal education	1.76 (.60)	1.64 (.71)	
Elementary education	1.76 (.57)	1.75 (.59)	
High school education	1.76 (.54)	1.76 (.57)	
Some college and college graduate	1.71 (.59)	1.69 (.59)	
Use of tobacco in any form			49.9 (<.001)
User	1.66 (.63)	1.16 (.92)	
Nonuser	1.80 (.54)	1.71 (.63)	

Knowledge score was computed by summing the correct responses to 2 questions on tobacco use and various forms of cancer.

Mean score: 1.73 (.61).

Range: 0–2; 0 (*N* = 2037); 1 (*N* = 2281); 2 (*N* = 19246).

**Table 3:** Tobacco consumption by gender, age, occupation, and educational levels.

Demographic variable	Male *N* (%)	Female *N* (%)	Total *N* (%)
Age			
18–34 years	1415 (35.8)	98 (26.1)	1513 (35)
35–44 years	948 (24.0)	89 (23.7)	1037 (24)
45–54 years	776 (19.7)	86 (22.9)	862 (20)
Above 55 years	810 (20.5)	102 (27.2)	912 (21)
Chi-square (*p*), df = 3	92.3 (<.001)	49.1 (<.001)	117.5 (<.001)
Occupation			
Not employed	610 (15.3)	289 (76.7)	899 (20.6)
Self-employed	3082 (77.3)	72 (19.1)	3154 (72.3)
Employed for wages	296 (7.4)	16 (4.2)	312 (7.1)
Chi-square (*p*), df = 2	97.6 (<.001)	363.6 (<.001)	2576.6 (<.001)
Educational levels			
No formal education	824 (20.7)	187 (49.6)	1011 (23.2)
Elementary education	1609 (40.3)	116 (30.8)	1725 (39.5)
High school education	1379 (34.6)	61 (16.2)	1440 (33.0)
Some college and college graduate	176 (4.4)	13 (3.4)	189 (4.3)
Chi-square (*p*), df = 3	244.3 (<.001)	14.5 (.002)	560.4 (<.001)

df: degree of freedom.

**Table 4:** Logistic regression: predictors of tobacco use.

Variable	Unadjusted odds ratio (95% CI)	Adjusted odds ratio (95% CI)	*p* values
Tobacco use (all forms) as the dependent variable			
Gender			
Female	Ref	Ref	
Male	14.08 (12.62, 15.71)	11.33 (9.78, 13.13)	<.001
Age			
18–34 years	Ref	Ref	
35–44 years	1.42 (1.30, 1.55)	1.74 (1.57, 1.93)	<.001
45–54 years	1.55 (1.42, 1.71)	2.21 (1.98, 2.47)	<.001
Above 55 years	1.39 (1.27, 1.52)	2.40 (2.14, 2.70)	.002
Occupation			
Self-employed	Ref	Ref	
Employed for wages	2.10 (1.79, 2.46)	2.16 (1.81, 2.58)	<.001
Not employed	0.17 (0.16, 0.18)	0.77 (0.69, 0.86)	<.001
Educational levels			
No formal education	Ref	Ref	
Elementary education	1.70 (1.56, 1.85)	1.74 (1.58, 1.92)	<.001
High school education	2.80 (2.60, 3.06)	3.00 (2.64, 3.33)	<.001
Some college and college graduate	2.75 (2.30, 3.29)	2.36 (1.91, 2.93)	<.001
Knowledge level	0.73 (0.69, 0.76)	0.60 (0.56, 0.63)	<.001
Smoking (cigarettes and bidis) as the dependent variable			
Gender			
Female	Ref	Ref	
Male	24.71 (21.06, 29.00)	24.32 (19.91, 29.70)	<.001
Age			
18–34 years	Ref	Ref	
35–44 years	0.65 (0.59, 0.72)	2.65 (2.37, 3.04)	<.001
45–54 years	0.55 (0.50, 0.61)	1.46 (1.33, 1.71)	<.001
Above 55 years	0.58 (0.53, 0.64)	1.15 (0.96, 1.26)	.09
Occupation			
Self-employed	Ref	Ref	
Employed for wages	6.59 (6.01, 7.22)	1.12 (1.00, 1.26)	.063
Not employed	0.85 (0.71, 1.02)	0.83 (0.68, 1.00)	.052
Educational levels			
No formal education	Ref	Ref	
Elementary education	0.60 (0.55, 0.66)	0.58 (0.52, 0.64)	<.001
High school education	0.42 (0.38, 0.47)	0.40 (0.35, 0.45)	<.001
Some college and college graduate	0.51 (0.41, 0.63)	0.56 (0.44, 0.72)	<.001
Knowledge level	1.20 (1.13, 1.27)	1.38 (1.29, 1.47)	<.001
Smokeless/chewing tobacco as the dependent variable			
Gender			
Female	Ref	Ref	
Male	15.14 (12.83, 17.87)	9.67 (7.87, 11.89)	<.001
Age			
18–34 years	Ref	Ref	
35–44 years	1.02 (0.92, 1.14)	1.30 (1.11, 1.53)	.001
45–54 years	0.76 (0.67, 0.86)	1.59 (1.34, 1.89)	.001
Above 55 years	0.48 (0.41, 0.55)	1.30 (1.09, 1.56)	.001
Occupation			
Self-employed	Ref	Ref	
Employed for wages	2.11 (1.78, 2.52)	1.82 (1.50, 2.21)	<.001
Not employed	0.16 (0.15, 0.18)	0.68 (0.59, 0.79)	<.001
Educational levels			
No formal education	Ref	Ref	
Elementary education	2.27 (2.00, 2.58)	1.69 (1.47, 1.94)	<.001
High school education	4.87 (4.28, 5.53)	3.34 (2.89, 3.87)	<.001
Some college and college graduate	6.06 (4.91, 7.48)	3.70 (2.91, 4.70)	<.001
Knowledge level	0.79 (0.74, 0.84)	0.69 (0.64, 0.74)	<.001

CI: confidence level.

Ref: reference category.

*Note*. Predictor variables included in logistic regression: gender, age, occupation, educational levels, and knowledge level.
